# Persistent Encephalopathy in a Noncirrhotic Patient: Do not Shun This Shunt

**DOI:** 10.14309/crj.0000000000000493

**Published:** 2020-12-08

**Authors:** Farah Kassamali, Steve Hu, Marina Roytman

**Affiliations:** 1 Department of Medicine, St. Mary's Medical Center, San Francisco, CA; 2 Department of Gastroenterology and Hepatology, UCSF Fresno, Fresno, CA

## Abstract

A portosystemic venous shunt is the formation of an abnormal connection between the portal vein and a systemic vein, allowing blood to bypass the liver. Portosystemic shunts are usually believed to be due to portal hypertension in the setting of underlying hepatic disease. We report a case of large, spontaneous intrahepatic portosystemic shunt in a noncirrhotic patient contributing to recurrent hepatic encephalopathy, also known as type B encephalopathy. Management of portosystemic encephalopathy involves occlusion of the shunt by endovascular management.

## INTRODUCTION

A portosystemic venous shunt is the formation of an abnormal connection between the portal vein and a systemic vein, allowing blood to bypass the liver. A large shunt can decrease hepatic reserve and eliminate the liver's ability to detoxify blood. Spontaneous formation of a portosystemic shunt is rare but can be described in patients. A study by Riggio et al determined that large shunts can abet persistent hepatic encephalopathy. In a cirrhotic patient with chronic hepatic encephalopathy and elevated ammonia without known liver dysfunction, large shunts should be investigated as a cause of neurocognitive abnormalities.^1,2^ We report a case of a large, spontaneous intrahepatic portosystemic shunt in a noncirrhotic patient contributing to recurrent hepatic encephalopathy.

## CASE REPORT

A 71-year-old woman with a medical history of nonalcoholic steatohepatitis, multiple episodes of gastrointestinal bleeding, deep venous thrombosis and pulmonary embolism requiring an inferior vena cava filter, and atrial fibrillation presented with recurrent episodes of altered mental status for over 2 years. Initially, her encephalopathy was attributed to repeated infectious insults and dehydration. However, because of persistent encephalopathy despite resolution of infection, an extended workup was performed including, but not limited to, thyroid function, vitamin levels, and dedicated cranial imaging—these were unremarkable. Ammonia levels were persistently elevated with a peak level of 310 μmol/L. Thus, hepatic encephalopathy was entertained as the presumptive diagnosis. This was further substantiated by her positive response to treatment with lactulose and rifaximin.

**Figure 1.**
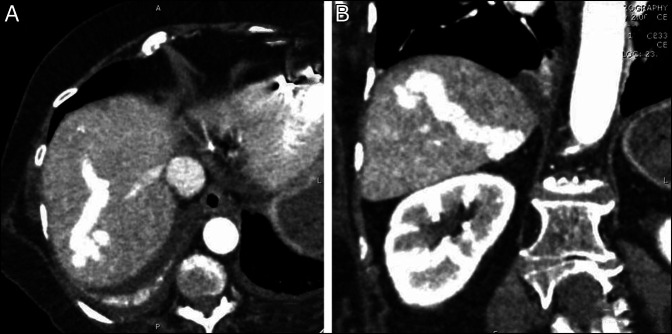
(A) Axial contrast-enhanced computed tomography demonstrating segment 7 large intrahepatic portovenous shunt and (B) coronal contrast-enhanced computed tomography demonstrating segment 5 large intrahepatic portovenous shunt.

Further diagnostic imaging was pursued because she did not have clinical features consistent with cirrhosis to contextualize hepatic encephalopathy. Dedicated computed tomography imaging revealed 2 large (12–14 mm) intrahepatic venous malformations manifesting as a portovenous shunt (Figure 1). On previous retrospective comparison imaging several years ago, this was appreciated as an incidental finding but less prominent. In addition, the magnitude of the shunt seemed to have contributed to capsular retraction resulting in nodularity of the liver.

**Figure 2.**
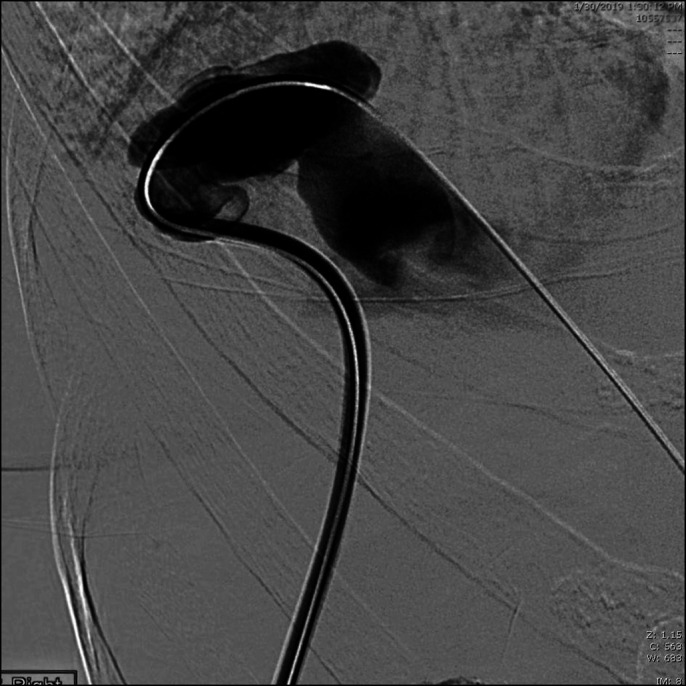
Transhepatic venogram with selective injection of the right posterior portal vein demonstrates portovenous shunt in segment 7 with massive hepatic vein dilation and lack of portal perfusion of liver.

**Figure 3.**
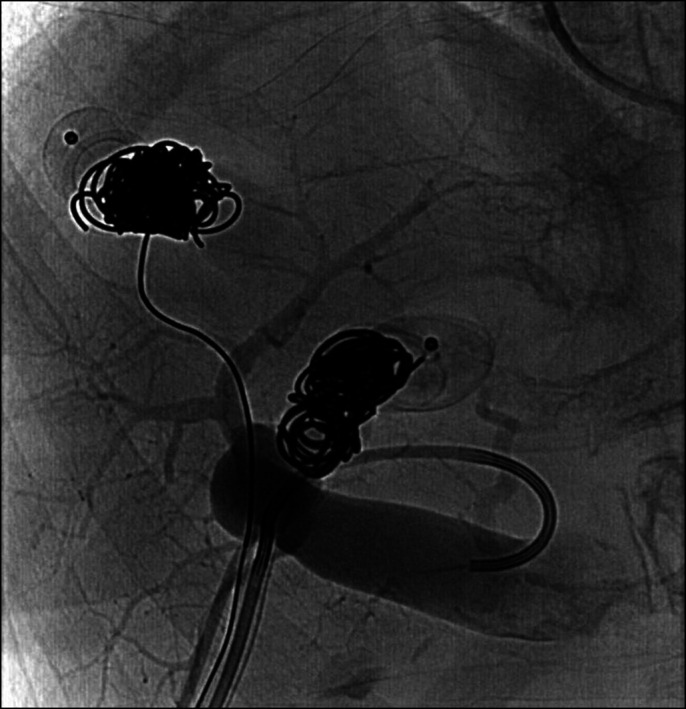
Transhepatic venogram postintervention with Amplatzer II 16 mm vascular plugs and adjacent Azur and Nester embolization coils of both portovenous shunts, now demonstrating restored portal vasculature.

Owing to repeated episodes of hepatic encephalopathy from portovenous shunting through her intrinsic hepatic venous malformation, the decision was made to undergo interventional coil embolization to close the anastomosis. Under ultrasound guidance, an enlarged portal vein in segment 7 was cannulated, and an 8-Fr vascular sheath was placed. A venogram was performed, confirming the large venous malformation with portovenous shunt (Figure 2). An Amplatzer II 16 mm plug was deployed, followed by the use of Azur and Nester embolization coils behind the plug. Postembolization angiography demonstrated absent flow through the shunt. Similar technique was used to intervene on the second large intrahepatic portovenous shunt in segment 5 and resulted in stagnant flow postembolization. A final main portal venogram demonstrated enhanced visualization of the hepatic parenchyma and no hepatofugal flow (Figure 3). On follow-up, the patient had no further episodes of hepatic encephalopathy and ammonia levels were normalized to 32 μmol/L.

## DISCUSSION

An abnormal connection between the portal and systemic circulation is known as a portosystemic shunt. These can be classified into extrahepatic (48.9%) or intrahepatic (36.2%).^2,3^ Portosystemic shunts are usually believed to be due to portal hypertension in the setting of underlying hepatic disease. Along with a comprehensive biochemical workup, various imaging modalities can assist in the detection and characterization of the anatomical anomaly, including ultrasound and cross-sectional imaging such computed tomography. Doppler ultrasonography allows visualization of abnormal blood flow and estimation of the shunt. Unmetabolized ammonia, a known culprit of hepatic encephalopathy, is highly dependent on portal flow and is elevated in the setting of a shunt.^4^

The prevalence of spontaneous portal shunts in cirrhotic patients is 38%–40% and is seen in 46%–70% of those with refractory encephalopathy.^5^ In a retrospective study with 2,000 patients, approximately 60% of cirrhotic patients had a spontaneous portosystemic shunt.^6^ In rare instances, spontaneous intrahepatic portosystemic shunts can be found in the absence of congenital abnormalities, trauma, or chronic liver disease. Although the etiology of shunt formation may not be clear, such venous malformations bypass the liver, causing unmetabolized portal blood to flow directly into the systemic circulation and subsequently cause persistent encephalopathy. Our patient presented with a portosystemic shunt within the right lobe of the liver. Previous cases hypothesize an embryonic explanation of shunts in the right lobe because of the communication between the omphalomesenteric venous system that empties into the sinus venosus.^7,8^

On the basis of having high ammonia levels, lack of advanced liver fibrosis, and the presence of a large intrahepatic shunt on imaging, the case is formally classified as portal-systemic encephalopathy (also known as type B encephalopathy) rather than traditional hepatic encephalopathy.^3,7,9^ In cases where recurrent portal-systemic encephalopathy is unable to be managed with conservative treatment, appropriate shunt closure using coils or surgical intervention must be considered.^3,9^ Most studied interventions are in cirrhotic patients and associated with improved survival, liver function, and prevention of hepatic encephalopathy.^10,11^ This case demonstrates successful angiographic embolization of a spontaneous intrahepatic portosystemic shunt, with confirmatory reduction in serum ammonia and restoration of normal hepatic portal circulation.

This is a unique case of a large spontaneous portosystemic shunt without underlying cirrhosis contributing to persistent portal-systemic encephalopathy. Hepatic encephalopathy associated with portosystemic shunting is known as type B encephalopathy, and occlusion of the shunt by endovascular management is the preferred treatment of choice. Providers should consider type B encephalopathy in patients with intractable presentations of hepatic encephalopathy.

## DISCLOSURES

Author contributions: F. Kassamali wrote the manuscript, reviewed the literature, and is the article guarantor. S. Hu provided the images. S. Hu and M. Roytman edited the manuscript and made critical comments and revised the manuscript for intellectual content. All the authors approved the final version of the manuscript.

Financial disclosure: None to report.

Previous presentation: This case was presented at the NCSCG Liver Symposium; December 7, 2019; San Francisco, California.

Informed consent was obtained for this case report.

## References

[1] Riggio O Efrati C Catalano C High prevalence of spontaneous portal-systemic shunts in persistent hepatic encephalopathy: A case-control study. Hepatology 2005;42:1158–65. 16250033 10.1002/hep.20905

[2] Qi X Ye C Hou Y Guo X A large spontaneous intrahepatic portosystemic shunt in a cirrhotic patient. Intractable Rare Dis Res 2016;5(1):58–60. 26989653 10.5582/irdr.2016.01000 PMC4761588

[3] Watanabe A Portal-systemic encephalopathy in non-cirrhotic patients: Classification of clinical types, diagnosis and treatment. J Gastroenterol Hepatol 2000;15:969–79. 11059925 10.1046/j.1440-1746.2000.02283.x

[4] Córdoba J New assessment of hepatic encephalopathy. J Hepatol 2011;54(5):1030–40. 21145874 10.1016/j.jhep.2010.11.015

[5] Wu W Han G Diagnosis and treatment of patients with cirrhotic portal hypertension and spontaneous portal shunt. J Clin Hepatol 2015;31(9):1528–31.

[6] Simón-Talero M Roccarina D Martínez J Association between portosystemic shunts and increased complications and mortality in patients with cirrhosis. Gastroenterology 2018;154(6):1694–705.e4. 29360462 10.1053/j.gastro.2018.01.028

[7] Raskin NH Price JB Fishman AA Portal-systemic encephalopathy due to congenital intrahepatic shunts. N Engl J Med 1964;270:225–9. 14072076 10.1056/NEJM196401302700503

[8] Park JH Cha SH Han JK Intrahepatic portosystemic venous shunt. Am J Roentgenol 1990;155:527–8. 2117349 10.2214/ajr.155.3.2117349

[9] Saad WE Portosystemic shunt syndrome and endovascular management of hepatic encephalopathy. Semin Intervent Radiol 2014;31(3):262–5. 25177088 10.1055/s-0034-1382795 PMC4139430

[10] An J Kim K Han S Lee J Lim Y Improvement in survival associated with embolisation of spontaneous portosystemic shunt in patients with recurrent hepatic encephalopathy. Aliment Pharmacol Ther 2014;39(12):1418–26. 24754260 10.1111/apt.12771

[11] Laleman W Simon-Talero M Maleux G Embolization of large spontaneous portosystemic shunts for refractory hepatic encephalopathy: A multicenter survey on safety and efficacy. Hepatology 2013;57:2448–57. 23401201 10.1002/hep.26314

